# The Impact of Cognitive Function on Virtual Reality Intervention for Upper Extremity Rehabilitation of Patients With Subacute Stroke: Prospective Randomized Controlled Trial With 6-Month Follow-up

**DOI:** 10.2196/33755

**Published:** 2022-07-08

**Authors:** Yan Leng, Wai Leung Ambrose Lo, Yu Rong Mao, Ruihao Bian, Jiang Li Zhao, Zhiqin Xu, Le Li, Dong Feng Huang

**Affiliations:** 1 Department of Rehabilitation Medicine First Affiliated Hospital Sun Yat-sen University Guangzhou China; 2 Guangdong Engineering and Technology Research Center for Rehabilitation Medicine and Translation Sun Yat-sen University Guangzhou China; 3 Department of Rehabilitation Medicine Seventh Affiliated Hospital Sun Yat-sen University Shenzhen China; 4 Institute of Medical Research Northwestern Polytechnical University Xi’an China

**Keywords:** stroke, motor function of upper extremity, virtual reality, cognitive function

## Abstract

**Background:**

Stroke is among the leading causes of long-term disability worldwide. Motor impairments after stroke not only impact the individuals quality of life but also lay substantial burdens on the society. Motor planning is a key component of cognitive function that impacts motor control. Hand movements such as grasping or reaching to grasp require the application of correct force and the coordination of multiple limb segments. Successful completion of hand motor task requires a certain degree of cognitive function to anticipate the requirement of the task. Cognitive function may thus be a confounding factor to rehabilitation outcomes.

**Objective:**

This study aims to explore the impact of cognitive function on functional outcomes in people with subacute stroke after VR intervention.

**Methods:**

Patients with stroke were first stratified into cognitively normal (CN) and cognitively impaired (CI), followed by allocation to the VR or control group (CG). Fugl-Meyer Assessment for Upper Extremity (FMA-UE), Barthel Index (BI), and Instrumental Activities of Daily Living (IADL) were recorded at baseline, 3 weeks after the intervention, and 3 and 6 months after the intervention. The between-group and within-group differences were assessed by repeated-measures analysis of variance (ANOVA).

**Results:**

The between-group comparison indicated that FMA-UE, BI, and IADL (time effect *P*<.001 for all) scores improved significantly in both groups after the intervention. Repeated-measures ANOVA indicated that FMA-UE, BI, and IADL (time effect *P*<.001 for all) were significantly different in each subgroup after the intervention. For BI score, the ANOVA results showed obvious interaction effects (treatment × time × cognitive effect, *P*=.04).

**Conclusions:**

VR intervention was as effective as traditional conventional therapy in improving upper limb function regardless of the cognitive functional level. Patients with stroke with impaired cognitive function may gain more improvement in upper limb function and independency in performing activities of daily living after a VR-based intervention.

**Trial Registration:**

Chinese Clinical Trial Registry ChiCTR-IOC-15006064; https://tinyurl.com/4c9vkrrn

## Introduction

Stroke is among the leading causes of long-term disability worldwide [1]. Despite the continuous improvement in rehabilitation technology, approximately 80% of acute and subacute stroke survivors continue to have residual upper extremity dysfunction of varying degrees [2]. Motor impairments after stroke not only impact the individuals quality of life but also lay substantial burdens on the society.

Virtual reality (VR) intervention is considered a promising approach in stroke rehabilitation. It is characterized by task-oriented and repetitive training with cognitive training elements [3,4]. VR systems create a simulated real life or imaginary environment where participants could interact dynamically [3,5]. Most published studies indicated that this technology provides a variable rehabilitation approach that improves physical function and reduces the demand on staff time [3]. A functional magnetic resonance image (fMRI) study previously published indicated cortical reorganization after VR training, which corresponded with upper limb motor function improvement [6]. Other studies also reported that the VR game system is superior to conventional therapy alone in improving upper extremity motor function recovery when used in conjunction with other interventions [3,7,8]. However, conflicting evidence was reported that VR intervention did not result in better function of the upper extremity when compared with traditional therapy [9,10], or that the change of the motor function was similar between the VR group and the control group [11,12]. A potential reason for the conflicting results may be related to the cognitive status of participants at baseline level.

Motor planning is a key component of cognitive function that impacts motor control [13]. It is known that cognitive function is a predictor of functional outcome in people with stroke [14]. Hand movements such as grasping or reaching to grasp require the application of correct force and the coordination of multiple limb segments [15]. Successful completion of hand motor task must therefore require a certain degree of cognitive function to anticipate the requirement of the task [16,17]. Thus, cognitive function may be a confounding factor of rehabilitation outcome. Studies that employed electro capnography to investigate the cognitive neural process of motor planning reported an increase in computational demand from bilateral hemispheres in patients with stroke [18,19]. A published study that utilized transcranial magnetic stimulation did not show direct evidence that the improvement in cortical activity is clinically relevant to upper limb function [20]. A potential reason is that transcranial magnetic stimulation (TMS) intervention has minimal cognitive involvement, which may limit its benefit on motor skills recovery. Initial studies in this area indicated that patients with higher cognitive status at the time of admission tended to have better rehabilitation outcomes [21,22]. By contrast, some other studies concluded that cognitive impairment had no negative effect on functional improvement [9,23]. Diamond et al [24] also proposed that the reason for poor functional outcome among patients with stroke having cognitive impairment may be more related to the low motor functional status at the time of admission, rather than cognitive impairment.

To date, it remains unclear if cognitive function may influence upper limb functional outcome in patients with stroke who undergo VR intervention and conventional therapy. VR intervention requires the capability of information identification and task execution during the training process [11,25]. This study aimed at exploring the impact of cognitive function on upper limb functional outcome after VR intervention in patients with subacute stroke.

## Methods

### Study Design

This was a prospective, single-blind, controlled trial, including 2 groups that were divided into 4 subgroups. Participants were first stratified into cognitively normal (CN) if the Mini-Mental State Examination (MMSE) score was greater than or equal to 27, and cognitively impaired (CI) if it was lower than 27 in accordance with previous studies [22,26,27]. Participants were then randomly allocated to either the VR intervention group or the control group, which gave a total of 4 subgroups: cognitively normal VR intervention group (CN_VR_), cognitively normal control group (CN_CG_), cognitively impaired VR intervention group (CI_VR_), and cognitively impaired control group (CI_CG_). Allocation sequence was randomly generated by a computer program.

### Participants and Recruitment

Participants were recruited from the inpatient ward between August 2008 and December 2017. Patients were screened for eligibility as part of routing assessment. Suitable participants were identified by the clinical team and given written information about the study. Participants who were interested to take part were asked to approach a member of the research team. Patients were included if they (1) had the first ever occurrence of unilateral cerebral infarction as confirmed by magnetic resonance imaging or computed tomography; (2) their initial onset was less than 6 months; (3) were able to sit independently for at least 30 minutes; (4) were able to complete the Chinese version of the MMSE assessment individually; (5) their education level is high-to-middle school (including technical secondary school) or above; (6) had no auditory and visual disorder; (7) were dextromanual; (8) were able to provide written informed consent. Patients were excluded if they had (1) uncontrolled medical conditions such as blood pressure or angina; (2) musculoskeletal impairments or fracture of the extremity; (3) a history of psychological conditions; or (4) were unwilling to participate in the study. Baseline information on anthropometric characteristics, including age, gender, hemiplegic side, and the Brunnstrom stage of upper extremity was collected prior to the experiment as part of routine clinical assessment.

**Figure 1:**
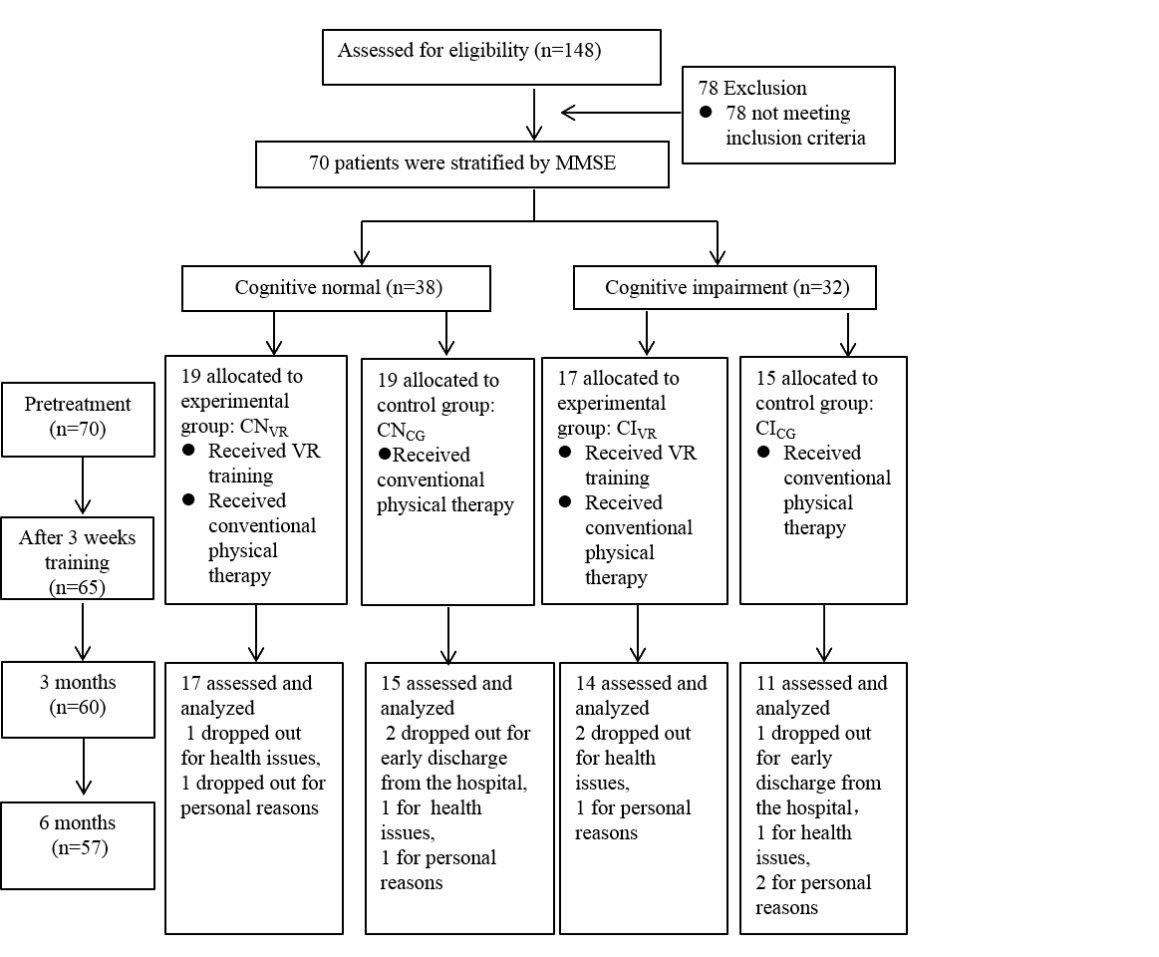
The number of participants at each stage of the study.

A total of 148 patients were screened for suitability, of which 70 patients met the inclusion criteria. As many as 38 and 32 participants were classified as cognitively normal and cognitively impaired, respectively; 13 participants did not complete the study due to personal reasons, health insurance issues, and unexpected early discharge from the hospital. Besides, 2 participants reported slight fatigue after VR training, and 1 complained of pain in shoulder, which resulted in the stopping of VR training immediately. Figure 1 illustrates the number of participants at each stage of the study.

### Intervention

#### Overview

The VR intervention group consisted of a 30-minute session of conventional rehabilitation program, followed by a 30-minute nonimmersive VR training using Microsoft Xbox 360 Kinect (Microsoft Corporation). Participants in the control group received 1-hour of conventional rehabilitation program. Both the VR group and the control group received treatment 5 days a week for a total of 3 weeks.

#### VR Intervention

This study used the Microsoft Xbox 360 Kinect to provide non-VR intervention. The motion camera monitors the body and extremity movements in 3D and tracks movements in real time. Participants were positioned 1.5-3 m away from the Kinect sensor. The following games were used for VR training: Balloon Buster, Table Tennis, Bowling, and Traffic Control. For participants who were at Brunnstrom stage 3 or above, bilateral shoulder and elbow movements were performed actively in the direction of abduction, adduction, flexion, and extension. For participants who were below stage 3 of the Brunnstrom classification, the unaffected arm may assist the activity of the affected arm. If the participant reported fatigue, abnormalities in breathing, or complained of pain, training was stopped immediately. Participants were informed about the experimental procedure. The operating procedure of the training device was demonstrated by the physiotherapist prior to start of the training session.

#### Conventional Therapy

The conventional physical therapy regimen for upper limb function included range of motion exercises, muscle strengthening, functional training, neurodevelopmental treatment, proprioceptive neuromuscular facilitation, and electrotherapy. The specific rehabilitation tasks for each participants were determined based on clinical needs as deemed appropriate by the treating clinicians.

### Outcome Measures

Outcome measures were recorded on 4 occasions: before the intervention, 3 weeks after the intervention, 3 and 6 months after the intervention. A licensed physiotherapist who was blinded to participants’ allocation and was not involved in the intervention program recorded the outcome measures at all measuring time points. Upper limb motor function was assessed by the Fugl-Meyer Assessment for Upper Extremity (FMA-UE) [28]. Barthel Index (BI) and Instrumental Activities of Daily Living (IADL) scales were adopted to assess the level of independence in ADLs [4,29]. All interventions were provided by clinical rehabilitation staff who were not blinded to group allocation.

### Statistical Analysis

Descriptive statistics were calculated to describe the data set of all variables. Independent *t* tests and chi-square tests were conducted to compare the demographic data and clinical characteristics between the VR group and the control group at baseline. Repeated measures analysis of variance (ANOVA) and the corresponding nonparametric test were conducted to compare the demographic data and clinical characteristics between the 4 subgroups at baseline. Repeated-measures ANOVA was used to compare the outcomes of FMA-UE, BI, and IADL scores of the 4 subgroups at different time points. The ANOVA results were adjusted using Bonferroni post hoc test if the interaction effects reach significant level (*P*=.05). The level of significance was set at an α level of .05. All statistical analyses were performed using SPSS version 22 (IBM, Inc.).

### Ethics Approval

The study was conducted at the Rehabilitation Department of a local hospital, and approved by the Medical Ethical Committee of the First Affiliated Hospital of Sun Yat-sen University (approval no.: 201488). All procedures were conducted in accordance with the Declaration of Helsinki. Participants were informed that they had an equal chance to be allocated to either the VR intervention or the conventional therapy group. Written consent was obtained from all participant prior to study enrollment. No changes were made to the planned methods after trial commencement. All mandatory laboratory health and safety procedures were complied with during the course of the study.

### Availability of Data and Materials

The data set supporting the conclusions of this article is available from the authors upon request.

## Results

Demographic characteristics of the patients in the VR group and the control group are presented in Table 1. No unintended effect was reported by participants of both groups. No statistically significant difference was also found between the VR group and the control group in terms of age (*P*=.85), stroke onset time (*P*=.10), MMSE score (*P*=.81), National Institute of Health Stroke Scale (NIHSS; *P*=.55) score, gender (*P*=.32), hemiplegic side (*P*=.44), and Brunnstrom stages of upper extremity (arm: *P*=.63; hand: *P*=.73). Table 2 presents the clinical characteristics of each subgroup. For NIHSS, the ANOVA showed significant interaction effects (treatment × cognitive effect *P*=.006). Bonferroni post hoc test indicated a significant difference between the CNVR subgroup and the CIVR subgroup (*P*=.004).

**Table 1:** Demographic characteristics of the VR group and the control group.

Variables	Experimental VR^a^ group (n=31)	Control group (n=26)	*P* value
Age (years), mean (SD)	59.25 (10.70)	59.12 (11.62)	.85
Disease duration (days), mean (SD)	43.42 (40.41)	30.15 (15.07)	.10
MMSE^b^, mean (SD)	25.32 (4.70)	25.00 (5.24)	.81
NIHSS^c^, mean (SD)	7.10 (3.29)	6.58 (3.25)	.55
Sex (female/male), n	6/25	8/18	.32
Hemiplegic side (right/left), n	13/18	14/12	.44
Brunnstrom stage of arm (stage 1-6), n	5/11/8/3/3/1	2/13/3/3/4/1	.63
Brunnstrom stage of hand (stage 1-6), n	5/16/2/4/2/2	5/11/2/2/5/1	.73

^a^VR: virtual reality.

^b^MMSE: Mini-Mental State Examination.

^c^NIHSS: National Institute of Health Stroke Scale.

**Table 2:** Demographic characteristics of 4 subgroups of the stroke survivors.

Variables	CN_VR_^a^ subgroup (n=17)	CI_VR_^b^ subgroup (n=14)	CN_CG_^c^ subgroup (n=15)	CI_CG_^d^ subgroup (n=11)	ANOVA^e^ (treatment)		ANOVA (cognitive)		ANOVA (treatment × cognitive)		Chi-square test	
					*F*_1,53_	*P* value	*F*_1,53_	*P* value	*F*_1,53_	*P* value	*χ* ^2^_3_	*P* value
Age (years), mean (SD)	57.29 (11.08)	62.57 (9.57)	54.40 (10.37)	65.55 (10.40)	<0.001	.99	8.653	.005	1.105	.30		
Disease duration (days), mean (SD)	36.47 (36.99)	51.86 (43.88)	28.80 (14.32)	32.00 (16.57)	2.656	.11	1.211	.28	0.521	.47		
MMSE^f^, mean (SD)	28.71 (1.05)	21.21 (4.06)	28.60 (1.12)	20.09 (4.57)	0.607	.44	102.844	<.001	0.416	.52		
NIHSS^g^, mean (SD)	5.35 (2.55)	9.21 (2.86)	6.87 (3.25)	6.18 (3.37)	0.901	.35	3.940	.052	8.070	.006^h,i^		
Sex (female/male), n	2/15	4/10	5/10	3/8							2.291	.54
Hemiplegic side (right/left), n	7/10	6/8	6/9	8/3							3.540	.32
Brunnstrom stage of arm (stage 1-6), n	1/5/4/3/3/1	4/6/4/0/0/0	1/10/1/2/1/0	1/3/2/1/3/1							18.683	.23
Brunnstrom stage of hand (stage 1-6), n	0/9/0/4/3/1	5/7/2/0/0/0	2/9/2/0/2/0	3/2/0/2/3/1							25.014	.050

^a^CN_VR_: cognitive normal virtual reality group.

^b^CI_VR_: cognitive impaired virtual reality group.

^c^CN_CG_: cognitive normal control group.

^d^CI_CG_: cognitive impaired control group.

^e^ANOVA: analysis of variance.

^f^MMSE: Mini-Mental State Examination.

^g^NIHSS: National Institute of Health Stroke Scale.

^h^ANOVA result was significant.

^i^Bonferroni post hoc test was significant between the CNVR subgroup and the CIVR subgroup (*P*=.004).

The group comparisons between the VR group and the control group indicated significant improvements in FMA-UE (treatment effect *P*=.67; time effect *P*<.001), BI (treatment effect *P*=.39; time effect *P*<.001), and IADL (treatment effect *P*=.97, time effect *P*<.001) in both the VR and control groups after the intervention (Table 3). Bonferroni post hoc test was not conducted to adjust the statistical result as no interaction effects were observed in ANOVA.

For the comparisons between the 4 subgroups, repeated measures ANOVA indicated that FMA-UE scores (time effect *P*<.001), BI scores (time effect *P*<.001), and IADL scores (time effect *P*<.001) were significantly different in each subgroup after the intervention. Table 4 presents the results of repeated measures ANOVA of the 4 subgroups. For FMA-UE and IADL scores, Bonferroni post hoc test was not conducted as the ANOVA results showed no interaction effects. For the BI score, the ANOVA results showed significant interaction effects (treatment × time × cognitive effect *P*=.04). Bonferroni post hoc tests indicated statistically significant differences between the CN_VR_ subgroup and the CI_VR_ subgroup at each measuring time point (Pretreatment *P*=.002; 3 weeks *P*=.005; 3 months *P*=.01; 6 months *P*=.03). There was also a statistically significant difference between the CI_CG_ subgroup and the CI_VR_ subgroup at baseline (*P*<.001).

**Table 3:** Clinical parameters of the experimental VR group and the control group before and after the intervention.

Parameter			Experimental VR^a^ group (n=31)		Control group (n=26)
**FMA-UE^b^ score^c^, mean (SD); (range)**					
	Pretreatment	27.68 (18.29); 4-63		27.69 (19.92); 6-65	
	3 weeks’ treatment	31.87 (19.42); 8-64		31.54 (19.96); 10-66	
	3 months	35.90 (21.19); 8-66		35.88 (19.95); 12-66	
	6 months	38.06 (21.48); 13-66		38.19 (19.01); 14-66	
**BI^d^ score^e^, mean (SD); (range)**					
	Pretreatment	50.65 (25.10); 10-100		60.77 (24.85); 20-100	
	3 weeks’ treatment	64.52 (26.86); 25-100		69.62 (24.41); 25-100	
	3 months	72.90 (22.67); 30-100		75.96 (23.20); 25-100	
	6 months	77.90 (20.55); 30-100		81.73 (19.95); 35-100	
**IADL^f^ score^g^, mean (SD); (range)**					
	Pretreatment	2.65 (1.99); 0-8		2.46 (2.13); 1-7	
	3 weeks’ treatment	3.16 (2.30); (0-8)		3.12 (1.97); 1-7	
	3 months	3.90 (2.53); 0-8		3.69 (2.33); 1-8	
	6 months	4.52 (2.94); 0-8		4.73 (2.28); 1-8	

^a^VR: virtual reality.

^b^FMA-UE: Fugl-Meyer Assessment for Upper Extremity.

^c^Two-way ANOVA: time, *P*<.001 (significant); treatment, *P*=.99; treatment × time, *P*=.95.

^d^BI: Barthel Index.

^e^Two-way ANOVA: time, *P*<.001 (significant); treatment, *P*=.34; treatment × time, *P*=.32.

^f^IADL: instrumental activities of daily living.

^g^Two-way ANOVA: time, *P*<.001 (significant); treatment, *P*=.92; treatment × time, *P*=.81.

**Table 4:** Clinical parameters of the CNVR, CIVR, CNVR, and CNCG subgroups before and after the intervention.

			CN_VR_^a^ subgroup (n=17)		CI_VR_^b^ subgroup (n=14)		CN_CG_^c^ subgroup (n=15)		CI_CG_^d^ subgroup (n=11)
**FMA-UE^e^ scores, mean (SD); range^f^**									
	Pretreatment	33.23 (18.76); 4-63		20.93 (15.79); 6-58		23.20 (17.65); 6-61		33.82 (22.03); 9-65	
	3 weeks’ treatment	39.47 (18.91); 8-64		22.64 (16.22); 8-58		26.07 (16.65); 11-60		39.00 (22.39); 10-66	
	3 months	43.58 (18.65); 14-66		22.64 (16.22); 8-58		31.47 (17.43); 12-66		41.91 (22.37); 12-66	
	6 months	46.35 (18.94); 14-66		28.00 (20.58); 5-65		34.20 (17.08); 14-66		43.63 (20.93); 14-66	
**BI^g^ scores, mean (SD); range^h^**									
	Pretreatment	63.24 (25.98); 25-100		35.35 (14.61); 10-60		57.33 (24.04); 20-100		65.45 (26.31); 25-100	
	3 weeks’ treatment	76.18 (23.15); 30-100		50.36 (25.83); 25-100		70.33 (23.94); 25-100		68.64 (26.18); 25-100	
	3 months	82.35 (17.42); 45-100		61.43 (24.37); 30-100		77.67 (20.78); 35-100		73.64 (27.02); 25-100	
	6 months	85.00 (17.68); 55-100		69.29 (21.83); 30-100		83.33 (20.59); 35-100		79.55 (19.81); 35-100	
**IADL^i^ scores, mean (SD); range^j^**									
	Pretreatment	3.82 (1.85); 2-8		1.21 (0.97); 0-3		2.80 (1.90); 1-7		2.00 (2.53); 0-7	
	3 weeks’ treatment	4.35 (1.90); 2-8		1.71 (1.90); 0-5		3.47 (1.88); 1-7		2.64 (2.16); 1-7	
	3 months	4.94 (1.87); 2-8		2.64 (2.71); 0-8		3.67 (2.09); 1-7		3.73 (2.83); 1-8	
	6 months	5.71 (2.23); 2-8		3.07 (3.12); 0-8		5.27 (1.94); 2-8		4.00 (2.68); 1-8	

^a^CN_VR_: cognitive normal virtual reality group.

^b^CI_VR_: cognitive impaired virtual reality group.

^c^CN_CG_: cognitive normal control group.

^d^CI_CG_: cognitive impaired control group.

^e^FMA-UE: Fugl-Meyer assessment for Upper Extremity.

^f^Repeated measures analysis of variance: Time, *F*_3,159_=52.398, *P*<.001 (significant); treatment, *F*_1,53_=0.099, *P*=.76; cognitive, *F*_1,53_=0.281, *P*=.60. Time × treatment, *F*_3,159_=0.013, *P*=.97; time × cognitive, *F*_3,159_=1.576, *P*=.22; treatment × cognitive, *F*_1,53_=7.358, *P*=.009 (significant). Treatment × time × cognitive, *F*_3,159_=1.329, *P*=.27.

^g^BI: Barthel Index.

^h^Repeated measures analysis of variance: Time, *F*_3,159_=49.619, *P*<.001 (significant); treatment, *F*_1,53_=1.446, *P*=.24; cognitive, *F*_1,53_=4.372, *P*=.004 (significant). Time × treatment, *F*_3,159_=1.687, *P*=.19; time × cognitive, *F*_3,159_=0.453, *P*=.66; treatment × cognitive, *F*_1,53_=4.111, *P*=.05 (significant). Treatment × time × cognitive, *F*_3,159_=3.161, *P*=.04 (significant). The Bonferroni post hoc test was significant between the CNVR subgroup and the CIVR subgroup (*P*<.001).

^i^IADL: Instrumental Activities of Daily Living.

^j^Repeated measures ANOVA: Time, *F*_3,159_=20.051, *P*<.001 (significant); treatment, *F*_1,53_=0.001, *P*=.98; cognitive, *F*_1,53_=11.807, *P*=.001 (significant). Time × treatment, *F*_3,159_=0.176, *P*=.87; time × cognitive, *F*_3,159_=0.808, *P*=.47; treatment × cognitive, *F*_1,53_=3.758, *P*=.06. Treatment × time × cognitive, *F*_3,159_=0.262, *P*=.80.

## Discussion

### Principal Findings

This study aimed to explore if cognitive status may influence upper limb functional outcomes in patients with stroke who underwent VR intervention and conventional therapy. This study observed no significant difference in all outcome measures between the VR intervention group and the conventional therapy group. The improvements in upper limb function and independency in performing activities of daily living persisted through to 3 and 6 months after the intervention in all subgroups. The improvement of BI scores in those with impaired cognitive function was higher than in those who were cognitively normal immediately after the VR intervention.

### Functional Activities

The results of this study indicated that participants with impaired cognitive function may gain more improvement in BI score upon undergoing VR intervention, especially 3 weeks after the intervention. VR intervention is a task-oriented tool with cognitive participation training. It requires participants to understand the task to be performed and respond accordingly in the virtual environment. It places more demand on movement anticipation and execution than conventional therapy [30]. The interactions between cognitive, motor function, and VR training are complex and are only partially understood in patients with stroke [7]. Oneş et al [31] reported a significant positive association between cognitive condition at the time of admission and functional outcomes at discharge, indicating the potential role of cognitive function in motor function recovery. Heruti et al [22] also reported that in addition to the positive correlation between cognitive status (assessed by the MMSE) and Functional Independence Measures (FIMs), participants with better cognitive function had shorter length of stay during hospitalization. When the effect of the VR intervention and conventional therapy in participants with impaired cognitive function was compared, the largest increase in BI score was observed in the CI_VR_ group from baseline to 3 weeks after the intervention. This may suggest that VR intervention may contribute to functional improvement at a faster rate than conventional therapy in patients with stroke with impaired cognitive function. A previous study that investigated the effect of VR intervention on cognitive function and lower limb function also reported greater improvements in cognitive abilities along with significant improvement in activities of daily living of the Functional Independence subscales, despite similar improvements in limb function observed between the VR group and the non-VR group [32]. These findings indicated that patients with stroke with cognitive impairment may gain more benefit from the VR intervention to improve functional activities. However, these benefits appeared to be limited to activities that were more related to basic self-care, rather than to the more complex functional activities that involved the interaction with the outside environment. This was supported by the results of our study that the largest improvement was observed in BI scores, rather than in IADL scores, in the 3 weeks after the intervention in the CI_VR_ group. BI and IADL are 2 fundamentally different aspects of functions. BI refers to functional ability to perform basic self-care activities such as toilet use, grooming, feeding, and walking, whereas the IADL scale assesses aspects such as transport, traveling, and social activities. Thus, any underlying impairments in motor and cognitive function may affect the performance of the IADL task to a greater degree than basic self-care activities [33].

### Upper Limb Motor Function

The mechanism of VR intervention was proposed to induce reorganization of the cerebral cortex [30,34,35]. An fMRI study published by our research group reported cortical reorganization of the contralateral sensorimotor cortex [6] after an intervention using Microsoft Xbox 360 Kinect. Positive outcomes for improving upper extremity motor function after the Microsoft Xbox 360 Kinect intervention were subsequently reported [9,29,36]. These findings are consistent with the data of our study, which also reported positive outcomes of upper limb motor function in the VR group. The group comparison between VR intervention and conventional therapy did not reveal significant differences in upper limb motor function and functional improvements. This finding is consistent with a published Cochrane review that concluded no significant difference between VR intervention and conventional therapy in upper limb functional outcome [3]. This finding, however, contradicts with some studies that reported VR intervention is superior to conventional therapy [3,37] in promoting upper limb functional recovery. A possible reason for the observed lack of significant difference between VR and conventional intervention was the matched intervention time in both groups. A Cochrane review reported that studies that reported superior outcome of VR tended to adopt VR as an augmentation to usual dosage of therapy where participants received more treatment time than the control group [3]. Thus, it could not be ruled out that the higher improvement may be related to the higher training dosage.

A previously published study proposed that cognitive status on admission correlated with motor outcome [22]. This study, however, did not observe any significant difference in FMA score after the intervention between participants who were cognitively normal or cognitively impaired at any of the recording time points. The findings of this study suggested that VR intervention is as effective as conventional therapy in improving upper limb motor function for patients with stroke who were cognitively impaired. These contradicting findings may be related to the diffused assessment of cognitive function of the MMSE [26] and the difference in the instrument adopted to assess motor function. MMSE broadly incorporates several categories such as time and place orientation, short-term memory, attention, recall, calculation, language, and visual spatial abilities [26], which may not be directly related to upper limb motor function. More recently published studies proposed that it was specifically the executive dysfunctions of cognitive impairment that may be related to motor function [38]. The study by Heruti et al [22] adopted the motor function component of the FIM and noted that the absolute gain of motor function may not be related to cognitive status, but is more related to the outcome of functional activities, which the FIM motor score was based on. This suggestion is given further support from a recent study that compared the effect of neurocognitive robot-assisted rehabilitation [39]. The study reported no significant difference in upper limb motor function (assessed by FMA-UE) and cognitive function (assessed by MMSE) gain was observed between neurocognitive robot-assisted rehabilitation and conventional therapy. This finding provide further evidence to suggest that cognitive status, as assessed by the MMSE, may not play an influencing role in the outcome of upper limb motor function provided by VR intervention [40].

### Limitations

The findings of this study should be interpreted with caution due to its limitations. There are insufficient data regarding the duration and frequency of treatment administered by the Microsoft Xbox 360 Kinect system. The training games were not specifically designed for stroke rehabilitation, which may contribute to the underestimation of the benefit of the VR intervention. The restricted hospitalization period (3 weeks) placed a limitation on treatment intensity. The sample size of each subgroup was small due to attrition during the follow-up period. The data therefore were likely to contain type II errors. Ikbali Afsar et al [11] proposed that the sensitivity of the Fugl-Meyer Scale could be affected by inadequate sample size, short duration of treatment, and short follow-up period. Future randomized trials with a large sample of subgroup population should be conducted to verify the findings of this study.

### Conclusion

In conclusion, VR-based intervention training is as effective as traditional conventional therapy regardless of cognitive status. Patients with stroke who have impaired cognitive function may gain more improvement in upper limb function that is related to self-care activity immediately after the VR intervention. Findings of this study adds further support that cognitive function plays an important role in upper limb motor function recovery. Clinicians who consider offering VR intervention for this pathological group should take into consideration the cognitive status to gain optimal benefit of the rehabilitation program. The presence of cognitive impairment should be considered when planning individualized rehabilitation program for patients with stroke to maximize upper limb function recovery.

## Appendix

Multimedia Appendix 1 CONSORT-EHEALTH checklist (V 1.6.1).

## Abbreviations

ANOVA: analysis of variance
BI: Barthel Index
CICG: cognitive impaired control group
CIVR: cognitive impaired virtual reality group
CNCG: cognitive normal control group
CNVR: cognitive normal virtual reality group
FIM: Functional Independence Measure
FMA-UE: Fugl-Meyer assessment for Upper Extremity
IADL: Instrumental Activities of Daily Living
MMSE: Mini-Mental State Examination
NIHSS: National Institute of Health Stroke Scale
TMS: transcranial magnetic stimulation
VR: virtual reality
